# Monkeypox in an immunocompromised patient with underlying human immunodeficiency virus and syphilis infections in Southern Florida of the United States: a case report

**DOI:** 10.1186/s12981-023-00504-4

**Published:** 2023-02-18

**Authors:** Wooyoung Jang, Likhita Kandimalla, Sivagami Rajan, Rafael Abreu, Jamie Enrique Campos

**Affiliations:** 1 School of Medicine, Hanyang University College of Medicine, 222-1 Wangsimni-ro, Seongdong-gu, Seoul, 04763 Republic of Korea; 2 School of Medicine, Gandhi Medical College, Secunderabad, India; 3 School of Medicine, Narayana Medical College, Nellore, Andhra Pradesh India; 4 Department of Family Medicine, Hialeah Hospital, Hialeah, FL USA; 5 Division of Infectious Disease, Department of Internal Medicine, Hialeah Hospital, Hialeah, FL USA

**Keywords:** Monkeypox, Acquired immunodeficiency syndrome, Human immunodeficiency virus infection, Syphilis

## Abstract

**Background:**

The orthopoxvirus causes the rare disease monkeypox, and underlying immune deficiencies might lead to worse outcomes. In this report, we described a rare case of monkeypox with an underlying immune deficiency caused by human immunodeficiency virus infection which was combined with syphilis. This report discusses differences in the initial clinical presentation and clinical course compared to typical monkeypox cases.

**Case presentation:**

We report the case of a 32-year-old man with human immunodeficiency virus infection who was admitted to a hospital in Southern Florida. The patient presented to the emergency department with shortness of breath, fever, cough and left-sided chest wall pain. Physical examination revealed a pustular skin rash, consisting of generalised exanthema with small white and red papules. Upon arrival, he was found to be in sepsis with lactic acidosis. Chest radiography showed left-sided pneumothorax and minimal atelectasis in the left mid-lung, with a small pleural effusion at the left lung base. An infectious disease specialist raised the possibility of monkeypox, and the lesion sample tested positive for monkeypox deoxyribonucleic acid. In this case, the possible diagnosis of skin lesions varied because the patient tested positive for syphilis and human immunodeficiency virus. For that reason, the differential diagnosis of monkeypox infection is prolonged owing to its initial atypical clinical features.

**Conclusions:**

Patients with underlying immune deficiency who have human immunodeficiency virus infection and syphilis can present with atypical clinical features and delay proper diagnosis, which can increase the risk of spreading monkeypox in hospitals. Thus, patients with rash and risky sexual behaviour should be screened for monkeypox or other sexually transmitted diseases such as syphilis, and a readily available, rapid, and accurate test is necessary to stop the spread of the disease.

## Background

The orthopoxvirus that causes monkeypox, belongs to the same genus as the variola virus, which causes smallpox, and the vaccinia virus, which is the virus used in smallpox vaccines [1, 2]. A rash resembling smallpox is caused by a zoonotic viral infection, known as monkeypox. However, compared to smallpox, monkeypox infections have much lower mortality rates and less person-to-person dissemination beyond households [1, 2]. Additionally, monkeypox rashes might resemble other infectious rashes that are seen more frequently, such as those caused by secondary syphilis, herpes simplex infection, and varicella-zoster virus infection [2].

Most monkeypox patients during the global outbreak in 2022 are symptomatic, and infections without symptoms are uncommon [3, 4]. The first case of monkeypox in 2022 was identified in Europe in May 2022, and cases associated with this outbreak have persisted and have been recorded in non-endemic nations worldwide, providing proof of community dissemination [2, 5]. The first instance of monkeypox in the United States was discovered on 17 May 2022 despite the patient's symptoms having been present for approximately two weeks before being confirmed with monkeypox [6, 7]. Numerous States in the United States have reported thousands of verified cases of monkeypox by July 2022 [1].

In this report, we present a rare case of monkeypox with an underlying immune deficiency caused by human immunodeficiency virus (HIV) infection which was combined with syphilis in Southern Florida. This report aims to discuss differences in the initial clinical presentation and clinical course, including symptoms, skin findings, and laboratory findings, compared to typical monkeypox cases.

## Case presentation

We report the case of a 32-year-old man who was admitted to a hospital in Southern Florida in July 2022. He was known to have HIV infection with a cluster of differentiation 4 (CD4) level of 185, was not compliant with his antiretroviral therapy, and had a drug abuse history positive for 3,4-methylenedioxy-methamphetamine (MDMA). The patient had no travel history 30 days before admission.

The patient presented to the emergency room with shortness of breath, fever, cough, and left-sided chest wall pain. The onset of symptoms occurred 5 days before, and the degree of symptoms was mild and constant. The patient denied any sore throat, nausea, or vomiting. Physical examination revealed a pustular skin rash, consisting of generalised exanthema with small white and red papules.

On arrival at the emergency room, he was found to be in a state of sepsis with lactic acidosis. He underwent chest radiography and was found to have 40% left-sided pneumothorax. Chest radiography also showed minimal atelectasis in the left mid-lung, with a small pleural effusion at the base of the left lung. He had sinus tachycardia with a heart rate of 101 beats/min, which returned to sinus rhythm after resorption of the pneumothorax. He tested negative for SARS-CoV-2 antigen and influenza A and B antigens. The patient was administered a single dose of azithromycin as an intravenous infusion and ceftriaxone. The patient was admitted to the hospital for further management.

**Fig. 1:**
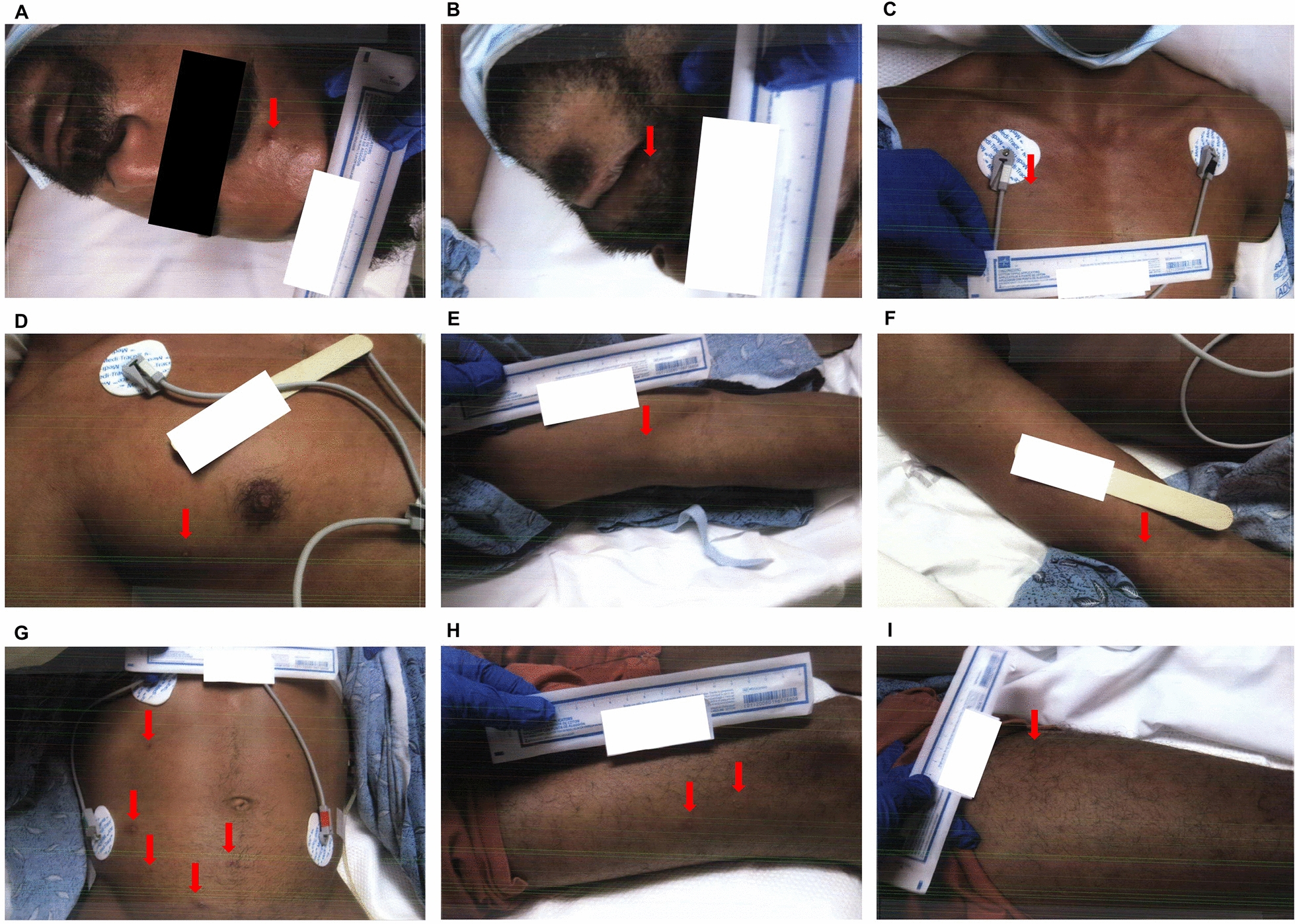
Skin lesions at admission day 1. **A** Face. **B** Bilateral lip. **C** Upper chest. **D** Right upper chest. **E** Right arm. **F** Right forearm. **G** Abdomen. **H** Right leg. **I** Left leg

Physicians from the departments of internal medicine, family medicine, infectious diseases, pulmonology, and critical care were involved in patient care. On day 1 of admission, the patient was afebrile. He was placed on a bi-level positive airway pressure (BiPAP) machine due to respiratory distress, insufficiency, and subjective weakness. The patient was shifted to the telemetry unit with contact and droplet precautions in view of skin lesions. The skin lesions were located on his face, lips, posterior neck, right hand, left antecubital area, left elbow, left forearm, right upper chest, back, right and left torso, abdominogenital region, buttocks, right posterior thigh, left thigh, bilateral lower legs, right inner ankle, and left medial foot. The lesions were multiple and scattered, characterised by macules, vesicles, and papules with dry scabs in some areas, associated with a moderate degree of pain, without any drainage, and with intact peri-wound areas (Fig. 1). The pain associated with the lesions resolved over the next 4 days. He was also diagnosed with generalised progressive macular hypomelanosis secondary to HIV infection. The laboratory investigations conducted on that day are presented in Table 1.

**Table 1:** The significant laboratory findings regarding HIV infection and syphilis

	Results
HIV-1 Antibodies	Reactive
HIV-2 Antibodies	Non-reactive
HIV-1/2 Interpretation	HIV-1 Positive
HIV-1 RNA quantitative viral load (copies/mL)	45,800
Log_10_ HIV-1 RNA (log copies/mL)	4.661
CD4 Helper absolute value (cells/μL)	185
CD4:8 Ratio	0.27
RPR Qualitative test	Reactive
RPR Titer	1:64
FTA-ABS	Reactive
Cryptococcal antigen	Negative

*HIV* human immunodeficiency virus, *RNA* ribonucleic acid, *Log* logarithm, *CD* cluster of differentiation, *RPR* rapid plasma reagin, *FTA-ABS* fluorescent treponemal antibody absorption

A new diagnosis of syphilis was made, and a monkeypox polymerase chain reaction (PCR) test was performed. The patient was started on oral antiretroviral drugs, including bictegravir, emtricitabine, and tenofovir, and continued to receive intravenous azithromycin and ceftriaxone.

On day 2 of admission, intravenous acyclovir injection was added to his treatment plan because of a skin rash associated with moderate pain. Chest radiography showed no interval change compared to the prior examination. Over the next few days, the patient became saturated with room air and was comfortable with no new acute symptoms, shortness of breath, or pain. The skin lesions appeared to be more vesicular, and some of them were crusting; hence, they were suspected to be shingles. The patient’s blood and sputum cultures were negative for any pathological organisms. Intramuscular injection of benzathine penicillin G was administered for syphilis.

**Fig. 2:**
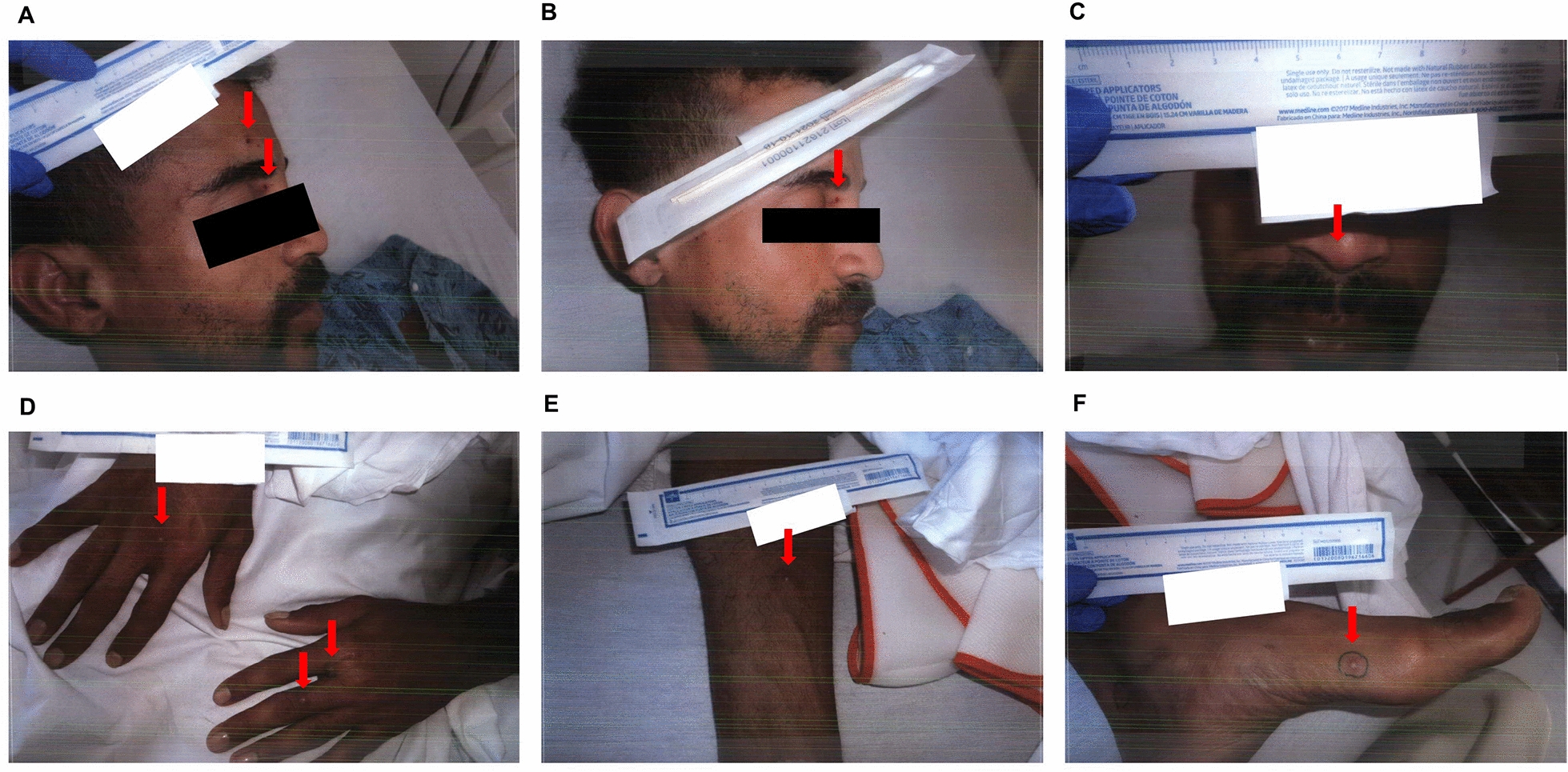
Skin lesions at admission day 3. **A**, **B** Face. **C** Nose. **D** Bilateral hands. **E** Right forearm. **F** Left foot

The following day (day 5), the results of the monkeypox PCR test were confirmed to be positive. The skin lesions appeared fragile, characterised by papules and vesicles, with no associated pain (Fig. 2). The patient continued to be in isolation with airborne, contact, and droplet precautions in place, and an order was placed for tecovirimat, a Food and Drug Administration (FDA)-approved drug for treating smallpox. He was additionally prescribed empirical oral sulfamethoxazole/trimethoprim. Azithromycin and acyclovir were then discontinued.

**Fig. 3:**
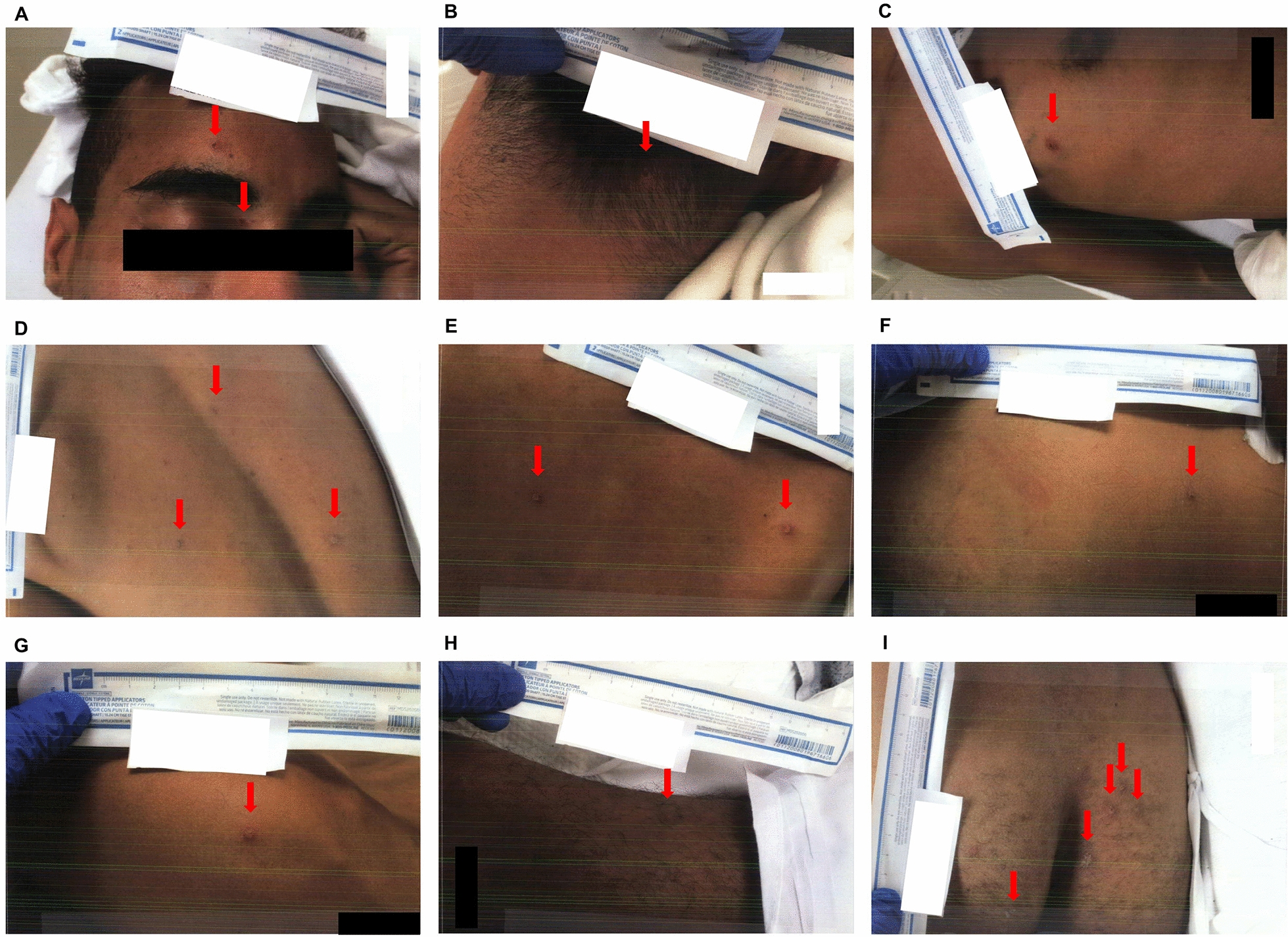
Skin lesions at admission day 5. **A** Face. **B** Posterior neck. **C** Right upper chest. **D**, **E**, **F** Back. **G** Right torso. **H** Left torso. **I** Sacrum and buttocks

**Fig. 4:**
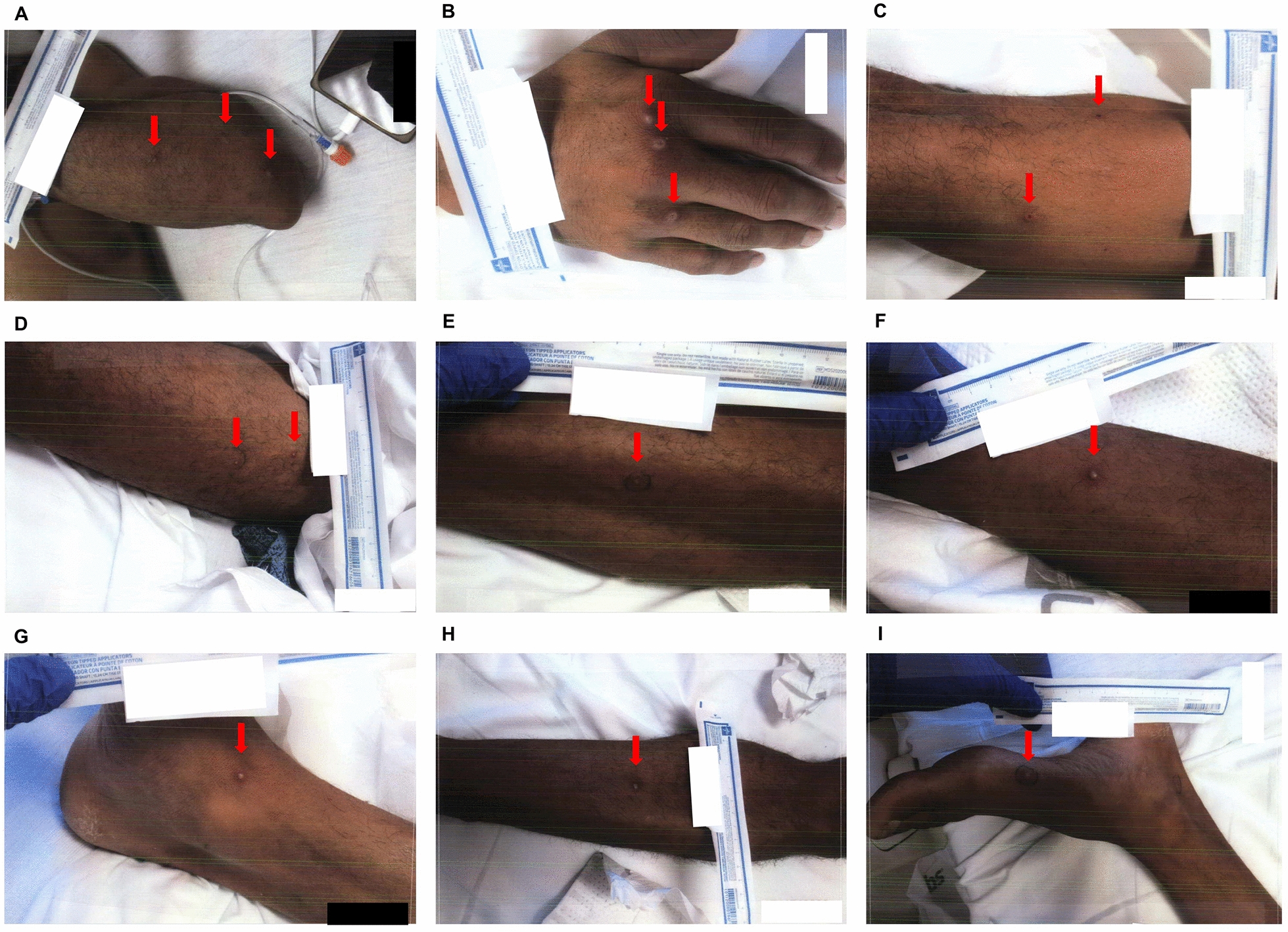
Skin lesions at admission day 5. **A** Left forearm. **B** Right hand. **C** Right thigh. **D** Left thigh. **E** Left lateral thigh. **F** Right leg. **G** Right inner ankle. **H** Left leg. **I** Left foot

Over the next few days, the patient’s skin lesions progressed to multiple lesions, disseminated with vesicular, pustulous character, and crusting was noticed at some sites (Figs. 3 and 4). In addition, he reported feeling better, and his physical examination findings were unremarkable. The patient was well saturated in room air, with oxygen supplementation as needed to maintain an oxygen saturation level above 92%. Another dose of benzathine penicillin G intramuscular injection was administered, but tecovirimat was yet to be administered.

On day 11, the patient was discharged from the hospital against medical advice. Upon leaving, the patient was instructed to keep himself covered and wear a mask. The patient’s vital signs were stable, and no drainage was noted in any of the lesions. The Infectious Diseases Department and the government were notified.

## Discussion

This report describes an immunocompromised patient diagnosed with human monkeypox. The patient initially presented with respiratory symptoms and skin lesions. However, the differential diagnosis of monkeypox infection is prolonged owing to its initial atypical clinical features. The possible diagnosis of skin lesions varied because the patient tested positive for syphilis and HIV. An infectious disease specialist raised the possibility of monkeypox, and the lesion sample tested positive for monkeypox deoxyribonucleic acid (DNA), which was detected by PCR. Monkeypox is currently spreading rapidly in the USA and Western Europe, and many cases are caused by sexual transmission between men who have sex with men [8]. As a result, it is anticipated that some individuals with monkeypox will also have other sexually transmitted infections, making the diagnosis of the condition challenging. Moreover, the likelihood of sexual transmission is evidenced by findings of primary genital and anal mucosal lesions, which may be the site of inoculation.

This clinical presentation is unique, as the patient also presented with respiratory manifestations suggestive of pneumothorax, along with skin lesions. The patient also had concomitant HIV and syphilis, and the atypical presentation might have been misdiagnosed as HIV-associated respiratory infection or sexually transmitted disease. In a previous study comparing the outcomes of monkeypox between HIV-negative cases and HIV-1 infected cases in Nigeria, significantly more HIV-1-positive individuals had secondary bacterial skin infections, genital ulcers, skin rashes ≥ 2 cm, and prolonged illness, and a higher mortality rate was found in HIV-1 infected group [9]. Therefore, monkeypox should be considered for at-risk patients.

There are no clear guidelines for the treatment of immunocompromised patients with monkeypox. In a previous retrospective observational study regarding the management of human monkeypox in the United Kingdom, one patient received tecovirimat 600 mg twice daily orally for two weeks, experienced no side effects, and had a shorter duration of illness and virus shedding than the other six patients who did not receive tecovirimat [10]. Tecovirimat was prescribed for this patient; however, he could not receive proper treatment for monkeypox. The current Centers for Disease Control and Prevention (CDC) guidelines suggest that there is no specific treatment for monkeypox infection. Tecovirimat, an antiviral drug, is recommended for severely immunocompromised patients. A smallpox vaccine has been shown to provide protection against monkeypox; however, its use has been limited to a few clinical trials. However, reports have suggested that most cases are self-limiting [11].

According to the CDC, Florida has the fifth-highest number of cases in the United States as of August 2022 [12]. Therefore, it is imperative that healthcare workers be educated regarding the clinical presentation and management of these cases. In addition, the follow-up of patients is essential to prevent transmission. Preventive strategies, including, but not restricted to, the isolation of cases and use of personal protective equipment (PPE), can go a long way in the battle against this disease.

## Conclusions

Patients with underlying immune deficiency who have HIV infection and syphilis can present with atypical clinical features and delay proper diagnosis, which can increase the risk of spreading monkeypox in hospitals. Thus, patients with rash and risky sexual behaviour should be screened for monkeypox or other sexually transmitted diseases such as syphilis, and a readily available, rapid, and accurate test is necessary to stop the spread of the disease.

## Data Availability

The datasets used and/or analysed during the current study are available from the corresponding author on reasonable request.

## Abbreviations

HIV: Human immunodeficiency virus
CD4: Cluster of differentiation 4
MDMA: Methylenedioxy-methamphetamine
BiPAP: Bi-level positive airway pressure
PCR: Polymerase chain reaction
FDA: Food and Drug Administration
DNA: Deoxyribonucleic acid
CDC: The Centers for Disease Control and Prevention
PPE: Personal protective equipment
